# lncRNA KCNQ1OT1 Promotes EMT, Angiogenesis, and Stemness of Pituitary Adenoma by Upregulation of RAB11A

**DOI:** 10.1155/2022/4474476

**Published:** 2022-04-07

**Authors:** Zuowei Li, Rong Ren, Lei Wang, Zhao Wang, Xin Zong, Ping Sun, Chunmei Zhu, Mingxia Guo, Guizhen Guo, Guo Hu, Ya'nan Wu

**Affiliations:** ^1^Shandong University of Traditional Chinese Medicine, Jinan, 250000 Shandong, China; ^2^Department of Encephalopathy, Affiliated Hospital of Shandong University of Traditional Chinese Medicine, Jinan, 250000 Shandong, China; ^3^Department of Traditional Chinese Medicine, People's Hospital of Chengyang, Qingdao, 266100 Shandong, China; ^4^Department of Oncology, Chengyang District Peoples Hospital, Qingdao, 266000 Shandong, China; ^5^Health Management Center, The Qingdao Affiliate Hospital of Shandong First Medical University, Qingdao, 266000 Shandong, China

## Abstract

This study is aimed at investigating the effect and mechanism of long noncoding RNA (lncRNA) KCNQ1OT1 on pituitary adenoma (PA). The KCNQ1OT1 expression in invasive and noninvasive PA tissues was detected by real-time fluorescence quantitative polymerase chain reaction (qPCR). The effects of KCNQ1OT1 on the proliferation of PA cells, namely, GH3 and HP75, were detected by CCK-8 experiment. The Transwell assay detected the effect of KCNQ1OT1 on the invasion of GH3 and HP75 cells. The effect of KCNQ1OT1 on the clonal formation ability was detected by clonal formation experiment. The double luciferase reporter assay and the miRNA pull down assay verified the binding of KCNQ1OT1 to miR-140-5p. Meanwhile, the regulatory effect of miR-140-5p on RAB11A was verified. qPCR results showed that KCNQ1OT1 was significantly increased in invasive PA compared with noninvasive PA tissues. Knockdown KCNQ1OT1 inhibited PA cell stemness, angiogenesis, and EMT. In addition, knockdown KCNQ1OT1 inhibited the proliferation, invasion, and clonal formation of PA. miR-140-5p is the target gene of KCNQ1OT1. miR-140-5p targets RAB11A directly. RAB11A can mediate the biological effects of KCNQ1OT1. Meanwhile, lncRNA KCNQ1OT1 can promote the EMT and cellular stemness of PA. Its mechanism of action is realized by inhibiting miR-140-5p. This result can provide a molecular basis for the further study of PA.

## 1. Introduction

A pituitary tumor is a common tumor of the central nervous system [1]. Pituitary adenomas (PA) account for approximately 15% of primary intracranial tumors [2, 3]. No drug is effective for PA. Radiation therapy remains controversial. The mechanism of the invasive biological behavior of PA remains to be studied further [4, 5]. The growth, infiltration, and metastasis of PA are associated with neovascularization. Effectively blocking tumor blood supply and inhibiting tumor angiogenesis can inhibit tumor growth.

Long noncoding RNA (lncRNA) is an endogenous transcribed RNA molecule. It is widely transcribed in the genome and lacks protein coding ability [6]. Increasing studies have shown that lncRNA participates in different biological processes through various mechanisms. Many changes in lncRNA expression levels, such as KCNQ1OT1 [7–11], may play an important role in the development of tumors. KCNQ1OT1 overexpression promotes the development and progression of hepatocellular carcinoma [11]. However, studies on the role of KCNQ1OT1 in PA are limited. MicroRNAS (miRNAs) are evolutionarily conserved noncoding RNAs [12]. They regulate posttranscriptional gene expression negatively by binding to complementary sequences on target mRNAs. miR-140-5p is an important tumor suppressor. The overexpression of miR-140-5p significantly reduced oral cancer [13], gastric cancer [14], colon cancer [15], and breast cancer [16, 17]. However, the effect of miR-140-5p on PA has been studied rarely.

Rab protein is a key regulator of cell membrane transport [18]. It plays an important role in the formation, transport, docking, and fusion of transport vesicles. Intracellular localization of different Rab proteins is coordinated during membrane transport, thereby forming a complex regulatory network. RAB11A plays a key role in regulating the formation and transportation of the recirculating endosome [19]. RAB11A directs the membrane vesicles that carry receptors to anchor on the plasma membrane, thereby realizing the recycling of receptors and lipids [20]. Any changes in the regulatory process may lead to abnormal protein transport, thereby leading to various diseases. At present, many reports on RAB11A gene are available, but reports on PA are few.

In this study, the expression levels of KCNQ1OT1 in invasive and noninvasive PA were detected to explore the role of KCNQ1OT1 in the occurrence, development, and invasion of PA. Meanwhile, the effect and mechanism of lncRNA KCNQ1OT1 on the proliferation and invasion of PA were also investigated. This study provides a theoretical basis for the diagnosis and treatment of PA.

## 2. Methods

### 2.1. Collection of Clinical Samples

A total of 45 noninvasive PA and 45 invasive PA diagnosed in our hospital from January 2018 to April 2021 were collected for analysis. All patients underwent endoscopic nasal surgery to remove PA. PA tissues were stored in liquid nitrogen within 30 min after operation. The diagnosis of a PA is confirmed by clinical presentation, hormone type, and magnetic resonance imaging (MRI) findings, as well as histopathological analysis and immunohistochemical staining of all pituitary hormones. The study did not include patients with prior radiation or recurrence. Invasive PA is defined as Hardy-Wilson class IV and/or Knosp classes III and IV. This study was approved by the Ethics Committee of Affiliated Hospital of Shandong University of Traditional Chinese Medicine and is in line with the Declaration of Helsinki. All patients' informed consent was obtained.

### 2.2. Cell Culture and Transfection

GH3 and HP75 cells were cultured in DMEM medium that contained 10% FBS (Gibco, Life Technologies, Rockville, MD, USA) (American Type Culture Collection, Manassas, VA, USA). These cells were cultured in an incubator at 37°C with 5% CO_2_. When 80% of the cells were fused, the cells were digested with 0.25% trypsin. Subculture was carried out in a ratio of 1 : 3. The number of cells was adjusted to 2 × 10^8^/mL and counted and inoculated into the corresponding culture plate. GH3 and HP75 cells were inoculated into a 6-well plate at a density of 1 × 10^5^ cells per well. When approximately 80% of the cells were covered, the culture medium was replaced with serum-free culture medium. Culturing was continued for 24 h. A total of 10 *μ*L of pcDNA3.1-KCNQ1OT1 or sh-KCNQ1OT1 and the control pcDNA3.1-NC or sh-NC was added to 50 *μ*L of serum-free medium. The mixture was mixed gently and let stand for 5 min at room temperature. A total of 2 *μ*L of liposomes and 5 *μ*L of the above mixed solution were mixed and let stand for 20 min. The mixture was shaken gently to mix and incubated at 37°C for 4–6 h. The serum-free medium was changed to complete medium and continue to culture for 24 h. Lipofectamine 2000 (Life Technologies, Rockville, MD, USA) was used as the transfection reagent. Cells were collected for subsequent experiments. miR-NC, miR-140-5p mimics, and anti-miR-140-5p were transfected simultaneously.

### 2.3. Cell Proliferation Assay

A total of 7000 cells were cultured in each well of a 96-well plate. The cells in each group were not transfected (blank group) as the control. The cell proliferation rate was measured according to the instructions of the CCK-8 cell activity detection kit (Beyotime, Shanghai, China). OD value at the wavelength of 490 nm was measured with a microplate reader. Cell proliferation rate = (OD of overexpression group − OD of blank group)/OD of blank group × 100%.

### 2.4. Clone Formation Experiment

A single cell suspension of PA was prepared, and the cell density was adjusted to 1 × 10^5^/mL. The suspension was diluted to 1 × 10^3^/mL. A total of 100 *μ*L of cell suspension was taken to inoculate a 6-well plate. A total of 2 mL of DMEM medium was added. The suspension was cultured in a constant temperature incubator at 37°C and 5% CO_2_ for 2 to 3 weeks. The colony formation was observed regularly, pictures were taken after staining and fixation, and the number of cell clones visible to the naked eye was counted. The results of the experiment were repeated 3 times for statistical analysis.

### 2.5. Immunofluorescence

The transfected cells were cultured for 48 h in a 6-well plate (covered with a cover glass) and taken out of the incubator. After washing with PBS, they were fixed with 4% paraformaldehyde for 20 min. The cells were treated with 0.5% Triton X-100 (Sigma-Aldrich, St. Louis, MO, USA) for 10 min. A total of 2% BSA was blocked for 1 h. A total of 50 *μ*L of diluted primary antibody was added dropwise to each coverslip and placed at 4°C overnight. About 50 *μ*L of diluted fluorescent secondary antibody was added to each cover glass and incubated for 1 h at room temperature. Next, dye DAPI was added to each well and placed at room temperature for 30 min. The sealing tablet was added to seal the tablet, and photos were observed and taken under a fluorescence microscope.

### 2.6. Transwell Experiment

The cells were inoculated into a 6-well plate at a density of 1 × 10^5^ cells per well. They were cultured in an incubator at 37°C with 5% CO_2_ for 24 h. After digestion and centrifugation, 2 × 10^4^ cells/mL serum-free medium was used to dilute the cells and prepare cell suspension. The cell suspension was added to the Transwell chamber (Millipore, Billerica, MA, USA) at 200 *μ*L per well. Meanwhile, 10% TBS and 500 *μ*L medium were added to Transwell. The medium was cultured in an incubator at 37°C with 5% CO_2_. It was removed after 24 h, and the excess liquid was sucked from the upper chamber of the Transwell. Crystal violet dye was added to the upper chamber. After 5 min, the dye was flushed with running water. Again, the cotton swab was gently rotated in the upper chamber to drain the water. A slide was placed on the microscope slide. The Transwell hole was placed upside down on top, and a picture was taken at 100x field of view count. The above experiment was repeated three times, and the average value was taken.

### 2.7. Real-Time Quantitative Reverse Transcription Polymerase Chain Reaction (qRT-PCR)

TRIzol (Invitrogen, Carlsbad, CA, USA) was used to extract total RNA according to product instructions. The RNA purity and concentration were determined using Nano-Drop 1000 (Thermo Scientific, Wilmington, DE, USA). The first-strand cDNA synthesis kit (Takara) was used to synthesize cDNA from total RNA (5 *μ*g). qRT-PCR was performed according to the instructions using the BioRad CFX-96 system and SYBR Green qPCR Super Mix-UDG kit (Takara). Next, a 25 *μ*L reaction system was set up. Amplification conditions were 50°C for 2 min, 95°C for 2 min, 95°C for 15 s, and 60°C for 30 s. A total of 40 cycles were completed. GAPDH was used as an internal reference. The primer sequence is the following: KCNQ1OT1, forward: 5′-GAUUCUCACUCGACACACGAU-3′, reverse: 5′-UCCGAGGCGUUGACCTAGAGC-3′; N-cadherin, forward: 5′-GGTGGAGGAGAAGAAGACCAG-3′, reverse: 5′-GGCATCAGGCTCCACAGTG-3′; Vimentin, forward: 5′-GAGAACTTTGCCGTTGAAGC-3′, reverse: 5′-GCTTCCTGTAGGTGGCAATC-3′; Snail, forward: 5′-CCTCCCTGTCAGATGAGGAC-3′, reverse: 5′-CCAGGCTGAGGTATTCCTTG-3′; Slug, forward: 5′-GGGGAGAAGCCTTTTTCTTG-3′, reverse: 5′-TCCTCATGTTTGTGCAGGAG-3′; E-cadherin, forward: 5′-TGCCCAGAAAATGAAAAAGG-3′, reverse: 5′-GTGTATGTGGCAATGCGTTC-3′; *β*-catenin, forward: 5′-ACAACTGTTTTGAAAATCCA-3′, reverse: 5′-CGAGTCATTGCATACTGTCC-3′; CD44, forward: 5′-TTGCAGTCAACAGTCGAAGAAG-3′, reverse: 5′-CCTTGTTCACCAAATGCACCA-3′; Oct4, forward: 5′-CTTGCTGCAGAAGTGGGTGGAGGAA-3′, reverse: 5′-CTGCAGTGTGGGTTTCGGGCA-3′; CD133, forward: 5′-TGGATGCAGAACTTGACAACGT-3′, reverse: 5′-ATACCTGCTACGACAGTCGTGGT-3′; CD166, forward: 5′-TCCTGCCGTCTGCTCTTCT-3′, reverse: 5′-TTCTGAGGTACGTCAAGTCGG-3′; SOX2, forward: 5′-GCCGATGTGAAACTTTTGTCG-3′, reverse: 5′-GGCAGCGTGACTTATCCTTCT-3; Nanog, forward: 5′-AATACCTCAGCCTCCAGCAGATG-3′, reverse: 5′-TGCGTCACACCATTGCTATTCTTC-3′; GAPDH, F: 5′-GGAGCGAGATCCCTCCAAAAT-3′, R: 5′-GGCTGTTGTCATACTTCTCATGG-3′. The relative mRNA level is calculated on the basis of the CT value (relative to the expression level of GAPDH) based on 2^-*ΔΔ*^CT.

### 2.8. Subcutaneous Tumor Bearing Experiment

The nude mice were purchased from Beijing Vital River Laboratory Animal Technology Co., Ltd. The logarithmic growth of PA cells was taken and digested with 0.25% trypsin and washed in serum-free medium. The cells were collected and counted. The concentration of living cells was adjusted to 5 × 10^7^/mL. Each nude mouse was inoculated with 0.2 mL/mouse at the soft skin of the right forelimb on the back. Each nude mouse was subcutaneously inoculated for 1 point. After four weeks, the experiment was over. The nude mice were sacrificed by carbon dioxide releasing devices. Considering the subcutaneous graft tumor was basically ovoid, the long diameter (mm) and short diameter (mm) of the tumor nodules were measured with a vernier caliper. Tumor volume was calculated by the following formula: volume (mm^3^) = length (mm) × width (mm)^2^/2. The long diameter and short diameter with a vernier caliper were measured at a regular time every day. The tumor volume curves of the subcutaneous transplantation in nude mice with tumors were plotted. Animal experiments are approved by the Ethics Committee of Affiliated Hospital of Shandong University of Traditional Chinese Medicine.

### 2.9. Immunohistochemical Experiment

The complete removal of the transplanted tumor tissue is called mass. The removed tumor tissue was fixed with 10% formalin. Conventional dehydration, paraffin embedding, and preparation of 4 *μ*m section were performed. Routine dewaxing to water was conducted. Immunohistochemical staining of KI-67 antibody (Abcam, Cambridge, MA, USA) markers was performed. The final concentration of KI-67 primary antibody was 1 : 100. The concentration of secondary antibody is 1 : 200. Hematoxylin was redyed. Nuclear staining was used as the exact positive staining criteria. Standard classification was the following: [1] Negative: no brown-yellow precipitate was observed in the cytoplasm, similar to the negative control, and no positive cells were found in the random field. [2] Weak positive: light brown-yellow reaction was evenly distributed in the cytoplasm, and the number of positive cells was <25%. [3] Moderate positive: deep brownish yellow reaction occurred in the cytoplasm, and the number of positive cells was 25%–50%. [4] Strong positive: brown-black reaction occurred in the cytoplasm, the cytoplasm was stained with coarse granules or blocks, and the number of positive cells was >50%. Five typical positive staining visual field areas were selected for each specimen, and their mean values were taken for analysis.

### 2.10. Double Luciferase Reporter Gene Detection Experiment

Using dual luciferase experiment, cells were transfected with miR-140-5p mimics and mimic-NC with psiCHECK2 empty, psiCHECK2-KCNQ1OT1-3′-UTR (WT), or psiCHECK2-KCNQ1OT1-3′-UTR (MUT). After 48 hours, the Promega dual luciferase reporter gene detection kit was used to detect firefly luciferase and Renilla luciferase. The relative activity of cellular luciferase was calculated. The ratio of firefly luciferase fluorescence intensity was compared with Renilla luciferase fluorescence intensity.

### 2.11. miRNA Pull Down

A biotin-labeled miRNA probe was used to pull down the mRNA of KCNQ1OT1 and RAB11A genes. First, miR-140-5p mimic (biotinylated miR-140-5p) modified with biotin was synthesized at the 3 ends. A random sequence was used as a control. The cells were transfected with the miR-140-5p probe, and the cells were harvested after 48 hours. Lysis buffer was added (20 mM Tris pH 7.5, 100 mM KCl, 5 mM MgCI_2_, 0.5% NP-40, and 1 U/*μ*L recombinant RNAse inhibitor). After lysis, centrifugation was conducted to remove cell debris. DNasel was added to the lysate to digest the DNA. After DNA digestion, the lysate was heated in a metal bath at 65° for 5 min. Then, it was quickly inserted into ice to cool. The lysate and avidin-coated magnetic beads (NEB) were mixed and incubated at 4° for 4 h. It was shaken gently with a shaker during incubation. After the incubation, the magnetic beads were washed twice with lysis buffer. TRIzol was used to extract RNA bound to magnetic beads for qRT-PCR analysis.

### 2.12. Statistical Analysis

SPSS V16.0 software (SPSS Inc., Chicago, IL, USA) was used for data analysis. Measurement data are expressed as the mean ± standard deviation. Independent sample *T*-test was used for the difference between the two groups. One-way ANOVA followed by Tukey's multiple comparison test was used for comparison between groups. Pearson correlation coefficient analysis is used for correlation analysis. *P* < 0.05 was considered statistically significant.

## 3. Results

### 3.1. KCNQ1OT1 Is Highly Expressed in the Tissues of Invasive PA

**Figure 1:**
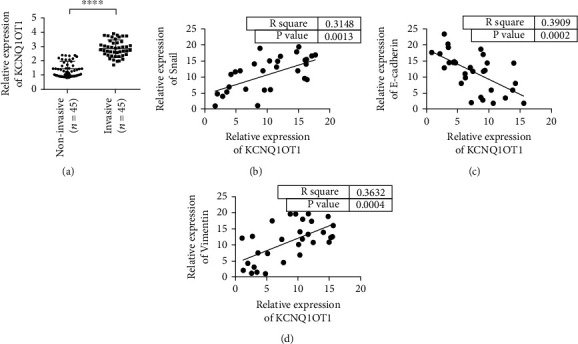
KCNQ1OT1 is highly expressed in aggressive pituitary adenomas. (a) The expression level of KCNQ1OT1 in invasive and noninvasive pituitary adenoma tissues is lower than that of adjacent tissues. (b) The expression level of KCNQ1OT1 is positively correlated with the coexpression of Snail. (c) The expression of KCNQ1OT1 is negatively correlated with the coexpression of E-cadherin. (d) The expression level of KCNQ1OT1 is positively correlated with the coexpression of Vimentin. ^∗∗∗∗^*P* < 0.0001.

We first detected the expression changes of KCNQ1OT1 in noninvasive and invasive PA tissues. The results showed that the expression level of KCNQ1OT1 in invasive tumor tissues was significantly higher than that in noninvasive tissues (Figure 1(a)). In addition, KCNQ1OT1 expressed a positive correlation with Snail (Figure 1(b)). The expression level of KCNQ1OT1 was negatively correlated with the coexpression of epithelial marker E-cadherin (Figure 1(c)). The expression of KCNQ1OT1 was positively correlated with the expression of mesenchymal marker Vimentin (Figure 1(d)).

### 3.2. Knockdown KCNQ1OT1 Can Inhibit the Cell Stemness and EMT of PA

**Figure 2:**
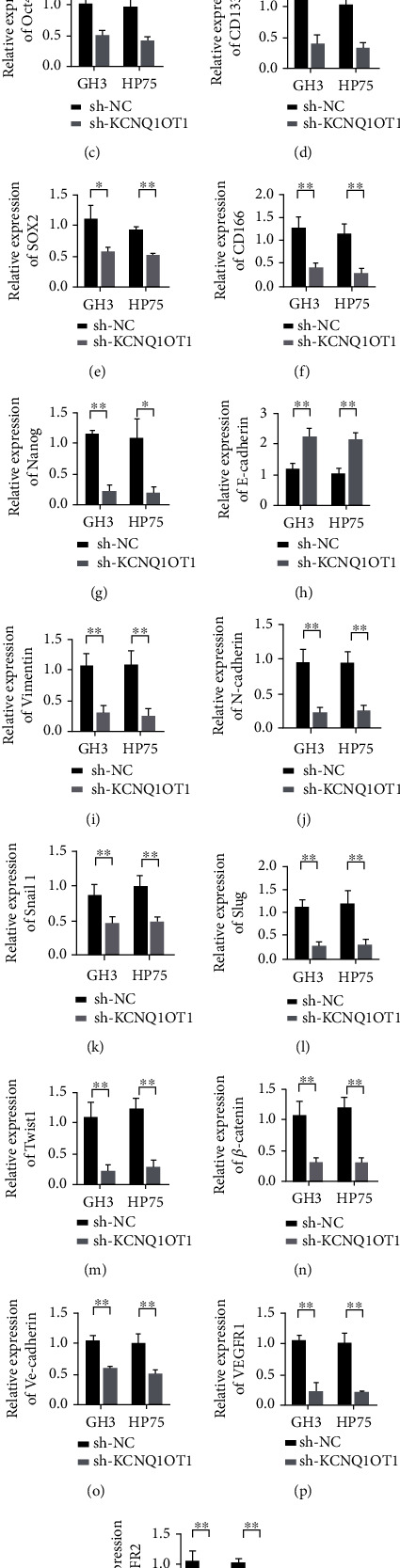
Knockdown KCNQ1OT1 inhibits pituitary adenoma cell stemness and EMT. (a) Validation of the efficiency of knocking down KCNQ1OT1 in GH3 and HP75 cells. (b) Detection of CD44 expression in GH3 and HP75 cells. (c) Detection of Oct4 expression in GH3 and HP75 cells. (d) Detection of CD133 expression in GH3 and HP75 cells. (e) Detection of SOX2 expression in GH3 and HP75 cells. (f) Detection of CD166 expression in GH3 and HP75 cells. (g) Nanog expression detection in GH3 and HP75 cells. (h) Detection of E-cadherin expression in GH3 and HP75 cells. (i) Detection of Vimentin expression in GH3 and HP75 cells. (j) Detection of N-cadherin expression in GH3 and HP75 cells. (k) Detection of Snail1 expression in GH3 and HP75 cells. (l) Slug expression detection in GH3 and HP75 cells. (m) Detection of Twist1 expression in GH3 and HP75 cells. (n) Detection of *β*-catenin expression in GH3 and HP75 cells. (o) Detection of VE-cadherin expression in GH3 and HP75 cells. (p) Detection of VEGFR1 expression in GH3 and HP75 cells. (q) Detection of VEGFR2 expression in GH3 and HP75 cells. ^∗^*P* < 0.05,  ^∗∗^*P* < 0.01.

In GH3 and HP75 cells, knockdown KCNQ1OT1 efficiency verification results showed that sh-KCNQ1OT1 plasmid could reduce the expression level of KCNQ1OT1 (Figure 2(a)). Further, we detected changes in the expression levels of CD44, Oct4, CD133, SOX2, CD166, and Nanog after knockdown of KCNQ1OT1. The results showed that knockdown KCNQ1OT1 could reduce the expression of CD44, Oct4, CD133, SOX2, CD166, and Nanog in GH3 and HP75 cells (Figures 2(b)–2(g)). In addition, changes in cellular transcription factors and EMT markers were also examined. The results showed that tapping KCNQ1OT1 could upregulate the expression of E-cadherin (Figure 2(h)). Meanwhile, tapping KCNQ1OT1 inhibited the expression of Vimentin and N-cadherin (Figures 2(i) and 2(j)). Further, we found that tapping KCNQ1OT1 inhibited the expression of Snail1, Slug, Twist1, and *β*-catenin (Figures 2(k)–2(n)). In addition, we analyzed the effect of KCNQ1OT1 on tumor vascular markers. qRT-PCR results showed that the expression levels of VE-cadherin, VEGFR1, and VEGFR2 were downregulated after KCNQ1OT1 was silenced (Figures 2(o)–2(q)). These results indicate that inhibition of KCNQ1OT1 can inhibit tumor angiogenesis.

### 3.3. Knockdown KCNQ1OT1 Can Inhibit the Proliferation, Invasion, and Clone Formation of PA

**Figure 3:**
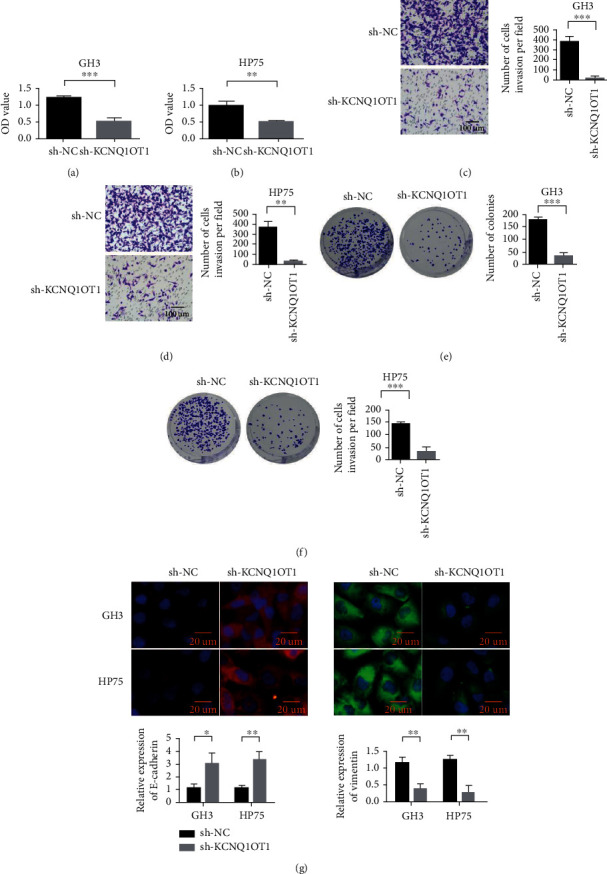
Knockdown KCNQ1OT1 inhibits pituitary adenoma proliferation, invasion, and colony formation. (a) Knockdown KCNQ1OT1 inhibits the proliferation of pituitary adenoma cell GH3. (b) Knockdown KCNQ1OT1 inhibits the proliferation of pituitary adenoma cell HP75. (c) Knockdown KCNQ1OT1 inhibits the invasion of pituitary adenoma cell GH3. (d) Knockdown KCNQ1OT1 inhibits the invasion of pituitary adenoma cell HP75. (e) Knockdown KCNQ1OT1 inhibits the cloning ability of pituitary adenoma cell GH3. (f) Knockdown KCNQ1OT1 inhibits the cloning ability of pituitary adenoma cell HP75. (g) Knockdown KCNQ1OT1 inhibits the expression of Vimentin in pituitary adenomas and promotes the expression of E-cadherin. ^∗^*P* < 0.05,  ^∗∗^*P* < 0.01, and^∗∗∗^*P* < 0.001.

To further verify the function of KCNQ1OT1 in PA cells, sh-KCNQ1ot1 and control vector sh-NC were transfected into GH3 and HP75 cell lines, respectively. The changes in cell proliferation, cell invasion rate, and cell clonal formation ability in different treatment groups were detected. At the same time, changes in EMT markers were detected. The results showed that the proliferation ability of GH3 and HP75 cells was reduced when the expression of KCNQ1OT1 was interfered compared with the control group (Figures 3(a)–3(b)). After knocking down KCNQ1OT1, the invasion rate of GH3 and HP75 cells decreased significantly (Figures 3(c) and 3(d)). Knockdown KCNQ1OT1 inhibited the clonal formation of GH3 and HP75 cells in PA cells (Figures 3(e) and 3(f)). In addition, knockdown KCNQ1OT1 inhibited the expression of Vimentin in PA and promoted the expression of E-cadherin (Figure 3(g)). It indicated that sh-KCNQ1OT1 inhibited the malignant behavior of GH3 and HP75 cells by inhibiting EMT.

### 3.4. Downregulation of lncRNA KCNQ1OT1 Expression Can Inhibit Tumor Growth In Vivo

**Figure 4:**
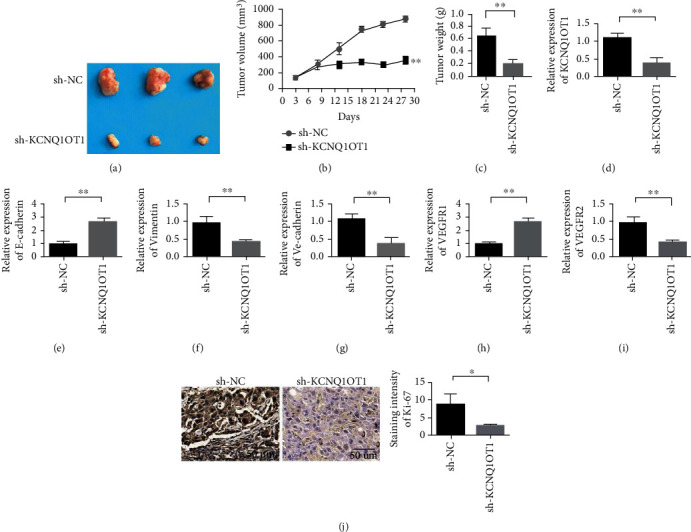
Downregulation of lncRNA KCNQ1OT1 expression can inhibit tumor growth in vivo. (a) Downregulation of lncRNA KCNQ1OT1 expression significantly inhibited the tumor growth of HP75 cells in nude mouse models. (b) Tumor volume detection. (c) Tumor weight detection. (d) Real-time PCR analysis showed that after knockout of lncRNA KCNQ1OT1, the expression of lncRNA KCNQ1OT1 in tumor tissues of nude mice was significantly reduced. (e) Real-time PCR analysis showed that after knocking out lncRNA KCNQ1OT1, the expression of E-cadherin in tumor tissues of nude mice was significantly increased. (f) Real-time PCR analysis showed that after knocking out lncRNA KCNQ1OT1, the expression of Vimentin in tumor tissues of nude mice was significantly reduced. (g) Real-time PCR analysis showed that after knocking out lncRNA KCNQ1OT1, the expression of VE-cadherin in tumor tissues of nude mice was significantly reduced. (h) Real-time PCR analysis showed that after knocking out lncRNA KCNQ1OT1, the expression of VEGFR1 in tumor tissues of nude mice was significantly reduced. (i) Real-time PCR analysis showed that after knocking out lncRNA KCNQ1OT1, the expression of VEGFR2 in tumor tissues of nude mice was significantly reduced. (j) Immunohistochemical analysis showed that lncRNA KCNQ1OT1 knockdown can significantly reduce the expression of KI-67. ^∗^*P* < 0.05,  ^∗∗^*P* < 0.01.

Compared with the sh-NC group, the downregulation of lncRNA KCNQ1OT1 significantly inhibited the tumor growth of HP75 cells in nude mice (Figures 4(a) and 4(b)). Tumor weight detection results showed that when KCNQ1OT1 was knocked down, tumor weight was significantly reduced (Figure 4(c)). Real-time PCR analysis showed that the expression of lncRNA KCNQ1OT1 in tumor tissues of nude mice was decreased significantly after lncRNA KCNQ1OT1 knockout (Figure 4(d)). Real-time PCR analysis showed that after lncRNA KCNQ1OT1 was knocked out, the expression of E-cadherin in nude mouse tumor tissues was significantly increased, whereas the expression of Vimentin in nude mouse tumor tissues was significantly decreased (Figures 4(e) and 4(f)). The expression levels of vascular markers VE-cadherin, VEGFR1, and VEGFR2 were also downregulated after KCNQ1OT1 was silenced in nude mouse tumor tissue (Figures 4(g)–4(i)). Immunohistochemical analysis showed that lncRNA KCNQ1OT1 knockdown could reduce KI-67 expression significantly (Figure 4(j)). This finding indicates that knockdown KCNQ1OT1 can inhibit the growth of PA.

### 3.5. Effect of Targeted Binding of KCNQ1OT1 on the Expression of miR-140-5p

**Figure 5:**
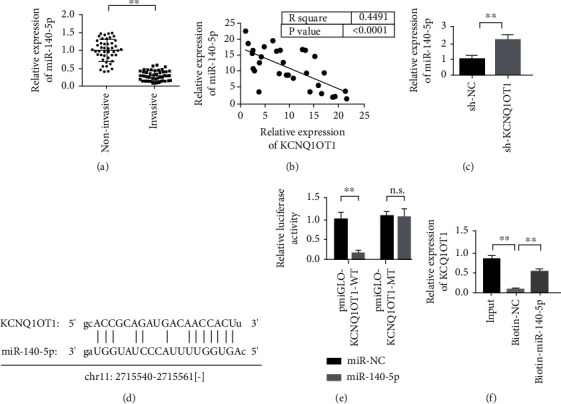
miR-140-5p is the target gene of KCNQ1OT1. (a) The expression of miR-140-5p is downregulated in aggressive pituitary tumor tissues. (b) The coexpression of miR-140-5p and KCNQ1OT1 is negatively correlated. (c) Knockdown KCNQ1OT1 upregulates the expression of miR-140-5p. (d) Schematic diagram of the binding site of miR-140-5p and KCNQ1OT1. (e) The dual luciferase reporter gene detection experiment verifies that miR-140-5p binds to KCNQ1OT1. (f) RNA pull down experiment verified the binding relationship between miR-140-5p and KCNQ1OT1. ^∗∗^*P* < 0.01.

The detection results of the expression level of miR-140-5p showed that miR-140-5p was downregulated in the invasive pituitary tumor tissues (Figure 5(a)). Meanwhile, miR-140-5p was negatively correlated with the coexpression of KCNQ1OT1 (Figure 5(b)). The experiment further verified that knockdown KCNQ1OT1 promoted miR-140-5p expression significantly (Figure 5(c)). Prediction software Starbase was used to predict that KCNQ1OT1 could target bind to miR-140-5p (Figure 5(d)). In addition, the double luciferase reporter assay further verified the binding of miR-140-5p to KCNQ1OT1 (Figure 5(e)). We further verified the binding relationship between miR-140-5p and KCNQ1OT1 through RNA pull down experiment. The experimental results showed that the miR-140-5p probe could enrich into KCNQ1OT1 significantly (Figure 5(f)). The above results indicated that KCNQ1OT1 could target bind to miR-140-5p, and sh-KCNQ1OT1 promoted the expression of miR-140-5p significantly.

### 3.6. In PA, miR-140-5p Directly Targets RAB11A

**Figure 6:**
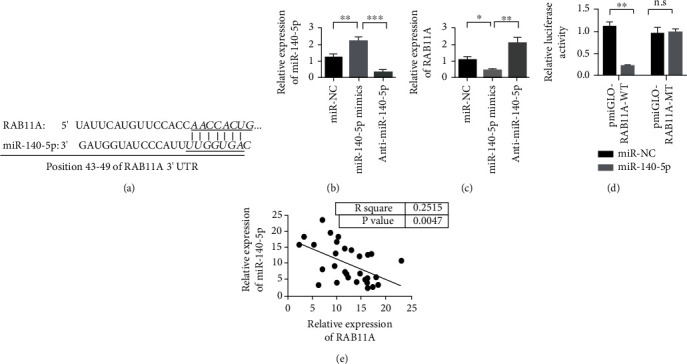
In pituitary adenomas, miR-140-5p directly targets RAB11A. (a) Schematic diagram of the binding site of miR-140-5p and RAB11A. (b) Verification of the transfection efficiency of miR-140-5p mimics and miR-140-5p inhibitor. (c) Overexpression of miR-140-5p inhibits the expression of RAB11A. (d) The dual luciferase reporter gene detection experiment verifies that miR-140-5p binds to RAB11A. (e) The coexpression of miR-140-5p and RAB11A is negatively correlated. ^∗^*P* < 0.05,  ^∗∗^*P* < 0.01, and^∗∗∗^*P* < 0.001.

The prediction software TargetScan was used to predict the ability of miR-140-5p to target RAB11A (Figure 6(a)). The experiment further verified that the overexpression of miR-140-5p inhibited the expression of RAB11A significantly. miR-140-5p was knocked down to promote the expression of RAB11A (Figures 6(b) and 6(c)). Double luciferase reporter gene detection experiment verified the binding of miR-140-5p to RAB11A (Figure 6(d)). At the same time, correlation analysis of coexpression of miR-140-5p and RAB11A showed that their coexpression was negatively correlated (Figure 6(e)).

### 3.7. RAB11A Expression Mediates the Biological Effect of lncRNA KCNQ1OT1

**Figure 7:**
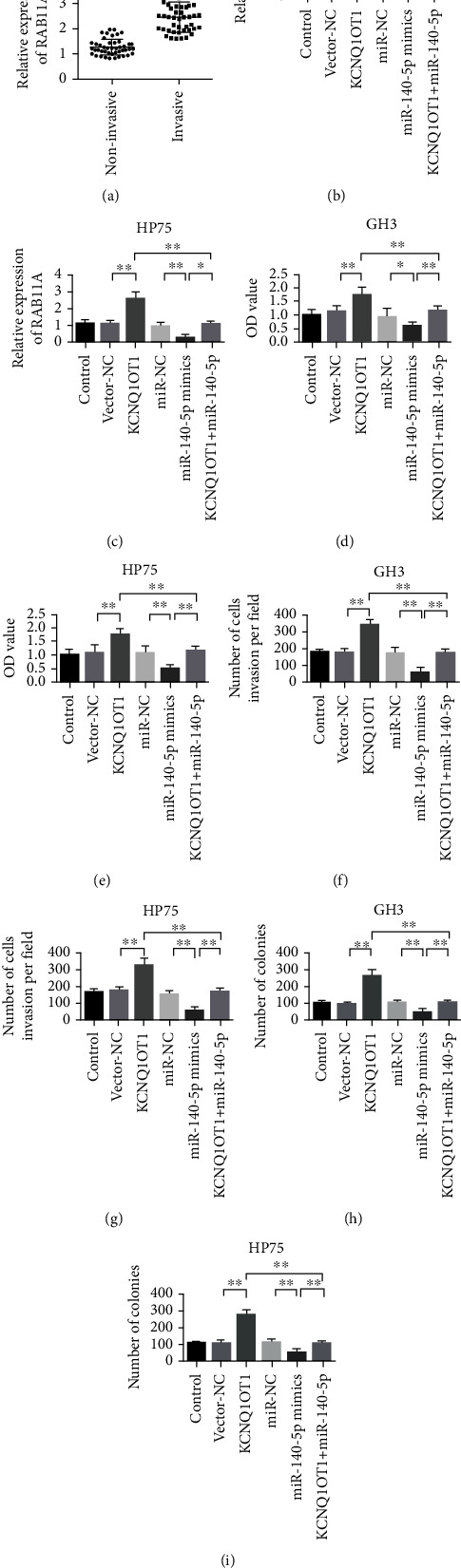
RAB11A expression mediates the biological effects of lncRNA KCNQ1OT1. (a) qRT-PCR detection of RAB11A expression in invasive pituitary adenoma tissue and noninvasive tissue. (b) Detection of RAB11A expression in GH3 cells after different treatments. (c) Detection of RAB11A expression in HP75 cells after different treatments. (d) GH3 cell proliferation detection after different treatments. (e) After different treatments, HP75 cell proliferation detection. (f) Detection of GH3 cell invasion after different treatments. (g) Detection of HP75 cell invasion after different treatments. (h) After different treatments, GH3 cell clone formation detection. (i) After different treatments, HP75 cell clone formation detection. ^∗^*P* < 0.05,  ^∗∗^*P* < 0.01, and^∗∗∗∗^*P* < 0.0001.

qRT-PCR was used to detect the expression level of RAB11A in invasive and noninvasive PA tissues. The results showed that RAB11A was upregulated in invasive pituitary tumor tissues (Figure 7(a)). After different treatments, RAB11A expression was detected in GH3 and HP75 cells, and the RAB11A protein expression was significantly increased after transfection with pcDNA3.1-KCNQ1OT1 by GH3 and HP75 cells compared with the control group. miR-140-5p mimic reduced the expression of RAB11A protein significantly. After the cotransfection of pcDNA3.1-KCNQ1OT1 and miR-140-5p mimic, the promoting effect of pcDNA3.1-KCNQ1OT1 on RAB11A expression was partially reversed (Figures 7(b) and 7(c)). The results of cell proliferation, invasion, and clonal formation showed that transfection with pcDNA3.1-KCNQ1OT1 significantly improved the proliferation, invasion, and clonal formation of GH3 and HP75 cells. The transfection of miR-140-5p mimics significantly inhibited the proliferation, invasion, and clonal formation of GH3 and HP75 cells. Compared with the pcDNA3.1-KCNQ1OT1 group, mimics of pcDNA3.1-KCNQ1OT1 and miR-140-5p mimic partially reversed the promoting effects of pcDNA3.1-KCNQ1OT1 on cell proliferation, invasion, and clonal formation (Figures 7(d)–7(i)).

## 4. Discussion

Pituitary tumors mainly originate from the anterior pituitary, accounting for about 10% of intracranial tumors [21]. Treating the disease is difficult because of its special growth location. So far, the pathogenesis of the disease is not very clear [22, 23]. No clear and uniform conclusion on its pathogenic gene is provided. Clinically, the complete removal of invasive PA is difficult with poor therapeutic effect and high recurrence rate, which are major problems in the treatment of PA [24, 25]. This study is aimed at describing the regulatory role of lncRNA KCNQ1OT1 in the RAB11A axis in PA through the “sponge” adsorption of miR-140-5p and describing the cellular/molecular mechanism of this axis function.

KCNQ1OT1 is overexpressed in many human tumors, including liver cancer [11], colon cancer [26], acute myeloid leukemia [27], and lung cancer [28]. KCNQ1OT1 can affect the invasion of cancer cells by regulating the EMT process related to cell invaders [29, 30]. KCNQ1OT1 was reported to be significantly more expressed in osteosarcoma than in adjacent tissues. KCNQ1OT1 can promote invasion, migration, and cell proliferation. In addition, KCNQ1OT1 can inhibit cell apoptosis [31]. It has also been linked to drug resistance, promotes the proliferation of TSCC in vivo and in vitro, and increases the resistance of TSCC. Qi et al. showed that knockdown KCNQ1OT1 inhibited cell invasion and sensitized osteosarcoma cells to CDDP by the upregulation of DNMT1-mediated Kcnq1 expression [32]. In this study, the KCNQ1OT1 level was upregulated in invasive PA. That is, the upregulation of KCNQ1OT1 expression contributes to the occurrence, development, and invasion of PA. Furthermore, KCNQ1OT1 overexpression significantly promoted the progression of PA. However, further research is still needed to confirm the conjecture of this study.

Analysis by predictive software showed that miR-140-5p was the downstream target gene of KCNQ1OT1. miR-140-5p has reportedly been identified as a human tumor suppressor. In this study, the expression of miR-140-5p in invasive PA was lower than that in noninvasive PA. The increase in miR-140-5p may be mediated by demethylation [33]. miR-140-5p, as the downstream target gene of KCNQ1OT1, targets and regulates the downstream RAB11A, and it is involved in the proliferation and tumor progression of PA. The results of this study further indicated that miR-140-5p inhibited the proliferation and invasion of PA. In this study, miR-140-5p inhibited the proliferation and invasion of PA, which was consistent with previous studies. miRNAs inhibit gene expression after target gene RNA transcription by acting on mRNA of target proteins, thereby affecting target proteins and related signaling pathways. The results of this study showed that RAB11A expression was low in miR-140-5p overexpressed cells. These results suggest that miR-140-5p can be involved in the pathophysiological process of pituitary tumors by regulating the expression of its target gene RAB11A in pituitary tumor cells. This study found that the expression of KCNQ1OT1 and miR-140-5p was negatively correlated in PA tissues. KCNQ1OT1 was upregulated in PA, while miR-140-5p expression was downregulated.

A large number of studies have demonstrated that the growth and metabolism of solid tumors require continuous vascular growth. Tumor angiogenesis is closely related to tumor growth, invasion, metastasis, grade, and prognosis. Tumor cells and interstitial blood vessels are interdependent. Tumor cells produce and secrete a series of angiogenic factors and inhibitory factors to regulate tumor angiogenesis, and tumor interstitial angiogenesis promotes tumor growth. Studies have shown that angiogenesis is associated with invasiveness in pituitary adenomas. In this study, inhibition of KCNQ1OT1 was found to inhibit tumor angiogenesis at the cellular level and animal tumor tissue level. Longatti and Tooze [34] investigated the proteins that contained the Tre-2/Bub2/Cdc16(TBC) domain and found that TBC1D14 was bound to the active form of RAB11 and participated in the early stages of autophagosome formation. RAB11 overexpression can inhibit autophagy. Previous studies confirmed that changes in RAB11A expression could affect the proliferation and apoptosis of tumor cells significantly. In addition, RAB11 overexpression promotes the proliferation of bladder cancer cells, which may be involved in the pathogenesis of bladder cancer as an oncoprotein. In this study, miR-140-5p targets RAB11A in PA directly. RAB11A expression mediates the biological effects of lncRNA KCNQ1OT1. As a target gene of miR-140-5p, RAB11A may mediate the promoting effect of the KCNQ1OT1/miR-140-5p axis on PA. KCNQ1OT1/miR-140-5p/RAB11A constitute the regulatory mechanism of ceRNA and affect the malignant progression of PA jointly.

## 5. Conclusion

This study revealed the differential expression of KCNQ1OT1 between invasive PA and noninvasive PA. The loss of KCNQ1OT1 expression may have a certain inhibitory effect against tumor response. In addition, the increased expression of KCNQ1OT1 was associated with the occurrence, development, and invasion of PA. KCNQ1OT1 is expected to be a target molecule for the treatment of PA. Predicting patients with a higher risk of progressing from PA to invasive PA is particularly important because of the poor prognosis in patients with invasive PA. The results of this study suggest that KCNQ1OT1 may be a potential diagnostic marker for invasive PA.

## Data Availability

The datasets used and/or analyzed during the current study are available from the corresponding author on reasonable request.
